# Exploring the Knowledge and Perception of Generic Medicines among Final Year Undergraduate Medical, Pharmacy, and Nursing Students in Sierra Leone: A Comparative Cross-Sectional Approach

**DOI:** 10.3390/pharmacy6010003

**Published:** 2018-01-04

**Authors:** Peter Bai James, Abdulai Jawo Bah, Emmanuel Kamanda Margao, Christian Hanson, John Alimamy Kabba, Shazia Qasim Jamshed

**Affiliations:** 1 Faculty of Pharmaceutical Sciences, College of Medicine and Allied Health Sciences, University of Sierra Leone, Freetown, Sierra Leone; jamepeb@yahoo.com (P.B.J.); emmiks@yahoo.co.uk (E.K.M.); 2 Faculty of Health, University of Technology Sydney, Sydney 2007, Australia; 3 Faculty of Basic Medical Sciences, College of Medicine and Allied Health Sciences, University of Sierra Leone, Freetown, Sierra Leone; abdulaijawobah@yahoo.com; 4 Pharmacy Board of Sierra Leone, Ministry of Health and Sanitation, New England Ville Freetown, Freetown, Sierra Leone; chrisson2001@yahoo.com; 5 Department of Pharmacy Administration and Clinical Pharmacy, Center for Drug Safety and Policy Research, Xi’an Jiaotong University, Xi’an 710061, China; 6 Directorate of Drugs and Medical Supplies, Ministry of Health and Sanitation, New England Ville Freetown, Freetown, Sierra Leone; fudimamy@yahoo.com; 7 Department of Pharmacy Practice, Kulliyyah of Pharmacy, International Islamic University Malaysia, Kuantan 25200, Malaysia

**Keywords:** generic medicines, knowledge, perception, medical students, pharmacy students, nursing students, Sierra Leone

## Abstract

Most low-income nations have national medicine policy that emphasized the use of generic medicines in the public health sector. However, the use of generics is often debatable as there are concerns over its efficacy, quality, and safety compared to their branded counterparts. This study was conducted to compare the knowledge and perception of generic medicines among final year undergraduate medical, pharmacy, and nursing students in Sierra Leone. We conducted a questionnaire-based cross-sectional study among these students at the College of Medicine and Allied Health Sciences University of Sierra Leone. Out of the 62 students, only two (2/62, 3.2%) knew about the acceptable bioequivalence limit. At least half of respondents in all three groups agreed that all generics are therapeutically equivalent to their innovator brand. At least half of the medicine (21/42, 50%) and nursing (6/9, 66.6%) students, compared to pharmacy students (5/11, 45.5%), believed that higher safety standards are required for proprietary medicines than for generic medicines. Most of them agreed that they need more information on the safety, quality, and efficacy aspects of generics (59/62, 95.2%). All three groups of healthcare students, despite variations in their responses, demonstrated a deficiency in knowledge and misconception regarding generic medicines. Training on issues surrounding generic drugs in healthcare training institutions is highly needed among future healthcare providers in Sierra Leone.

## 1. Introduction 

A huge proportion of health budget of most developing countries goes to purchasing drugs needed to treat diseases of public health importance [1]. Therefore, access to treatment is challenged by the availability of affordable medicines. One-third of the population in the developing world does not have access to medicines [1]. Accessibility is often hampered, among others, by high prices of these drugs [2]. As a strategy to increase access, most developed and developing countries have instituted measures to reduce expenditure on medicines. One such approach involves promoting the use of generic medicines among healthcare providers [3].

In Sierra Leone, even though there is no particular policy on generic substitution, the national drug policy does state that all prescription and dispensing of medicines in the public sector should be done using generics. The policy further states that the Ministry of Health and Sanitation (MOHS) will promote generic prescribing and dispensing in the private sector [4]. However, the implementation of such policy statements remains a challenge in promoting rational medicine use. With patient out-of-pocket expenses accounting for 62% of healthcare costs [5], access to, and the utilization of, health services including pharmaceuticals among the general population is still poor. On the other hand, health service utilization including pharmaceuticals seems to have improved with the passage of the free health care policy for pregnant women, lactating mothers, and children under five [6]. This initiative ensures that medicines prescribed and dispensed are from the essential drug list all of which are listed by their generic names [7]. For example, 71% of medications prescribed for children under five at the tertiary pediatric hospital were generics [8]. Despite such improvement, anecdotal reports suggest that the use of generics is still a controversial issue among health care providers as there is a huge concern over their efficacy, quality, and safety compared to their branded counterparts. Similar misconceptions about generics were reported elsewhere among future health care professionals [9,10]. 

Studies elsewhere have indicated that it is often a challenge to change perception as well as the prescribing and dispensing behavior of healthcare providers about generics [11,12,13]. Therefore, the need for education to change attitudes is required especially for future health care providers as their current perception of generic medicine can impact their future prescribing and generic substitution pattern. Such interventions have been recommended in the developed world and in some developing countries as a way to promote the use of generics [14]. However, studies on knowledge and perception of generic drugs among healthcare providers and students are few, especially in the African region. Currently, the knowledge about and perception of generic medicines among health care providers and students in Sierra Leone are unknown. To address this gap in knowledge, this study compares the knowledge about and perception of generic medicines among final year medical, pharmacy, and nursing students in Sierra Leone.

## 2. Materials and Methods

The present study was conducted among final year undergraduate medical, pharmacy, and nursing students at College of Medicine and Allied Health Sciences University of Sierra Leone (COMAHS-USL) using a cross-sectional study design. Sixty-eight final year students (46-medical, 11-pharmacy and 11-nursing) registered for the 2014/2015 academic year constituted the study population. We conducted the study between June and August 2015. COMAHS-USL is the only tertiary institution in Sierra Leone that offers undergraduate programs in medicine, pharmacy, and nursing [15]. The undergraduate medical and pharmacy programs have been in existence since the inception of the college in 1988 while the nursing program started in 2005 when the national school of nursing became part of COMAHS-USL [15]. The medicine, pharmacy, and nursing programs run for six, five, and four years, respectively. Due to the stringent entry requirements and cost, admission rates in to these programs are usually low. The current medicine, pharmacy, and nursing curricula do offer an overview of generic bioequivalent and rational medicine use that is taught as part of the general pharmacology module in the third professional year. This module has three contact hours per week and is taught for two months [16]. However, for pharmacy students, these topics continue to be extensively covered in the fourth and final professional years under their clinical pharmacy and pharmaceutics courses [16]. These courses have three contact hours per week and are taught for three months [16]. We sought ethical approval from the COMAHS-USL Research and Ethics Committee before the study commenced. 

A validated questionnaire used in a Malaysian study [17] was used to collect data from participants in this study. Some modifications were made to fit our local context. For example, the original validated questionnaire asked respondents to indicate their ethnicity since Malaysia is host to different ethnic groups. We decided to remove the question on ethnicity since Sierra Leone is homogeneous with regard to ethnicity. The questionnaire consisted of three sections. The first section looked at respondents’ demographics such age, sex, and course of study. The second section consisted of 13 items that attempt to assess students’ knowledge about generic and their branded name medicine, 12 of which were evaluated using a five-point Likert scale. The remaining item specifically asked about the allowable regulatory bioequivalence limits that the Pharmacy Board of Sierra Leone (PBSL) stipulates when comparing generic medicines and their branded counterparts. We included the following statements to aid their understanding of what bioequivalence is: “In pharmacology, the term bioavailability refers to how fast and how much a drug is absorbed and becomes available at the site of the drug action”; “The PBSL requires that a generic medicine has a comparable bioavailability to that of its proprietary counterpart.” The third section consisted of seven items that seek to understand respondents’ perceptions about generic and brand medicines using five-point Likert scale responses. Strongly agree, agree, neutral, disagree, and strongly disagree were considered as point descriptors. Written consent was solicited after the rationale of the study was explained to participants. Questionnaires were distributed to consenting students in their classes and returned within 20–25 min to ensure a high response. Students were told that they were at liberty to opt out of the study at any time while completing the questionnaire. They were also assured that they would not be penalized whatsoever if they decide not to participate. It was also clearly stated on the consent form that the questions asked were for research purpose only and it was not going to be part of any assessment required for them to graduate. Students were not allowed to use any reference to answer the questions. Paper-pencil format was used as the method for questionnaire administration. All filled questionnaire were collected and checked for completeness.

Analysis of the data collected was done using SPSS version 16 (SPSS, Inc., Chicago, IL, USA). Descriptive statistics were used to calculate frequency counts and percentages for respondent demographic characteristics and Likert scale responses. Likert scale responses “strongly agree and agree” were grouped as a positive response, while “strongly disagree and disagree” were grouped as a negative response when interpreting the results. Associations between these dependent variables (all knowledge and perception statements) and independent variable (program of study) were computed using the Kruskal–Wallis test. This test was used due to the small size of the study population (*n* = 68) and the skewness of the population in each cadre. An alpha value less than 0.05 was considered statistically significant for differences observed among the three groups.

## 3. Results

### 3.1. Respondent Demographics

The overall response rate was 91.2% (62/68). Most of the students were male (62.9%), between the age of 20–29 years, and from the faculty of medicine (Table 1).

### 3.2. Knowledge of Regulatory Bioequivalence Limit among Final Year Undergraduate Medicine, Pharmacy, and Nursing Student

**Figure 1:**
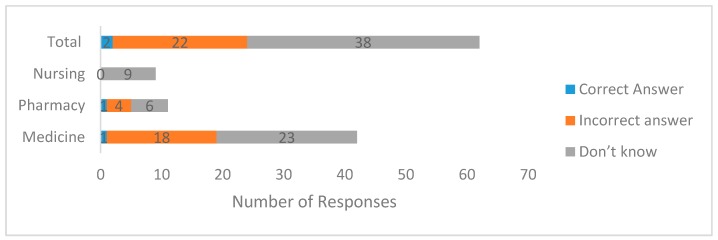
Knowledge of regulatory bioequivalence limit among final year undergraduate medicine, pharmacy, and nursing students. The Kruskal–Wallis test showed no statistically significant difference (*p* = 0.545) among the three groups of final year undergraduate students.

On their knowledge of regulatory bioequivalence limit, students were asked to choose the correct allowed bioequivalence limits when comparing a generic medicine with an innovator branded product. Six options were presented from which they were asked to select the correct answer. The allowed bioequivalence limit is 80–125%. We grouped their responses into three categories: “correct,” “incorrect,” and “I do not know.” Based on their responses, only two students chose the correct answer (2; 3.2%), while the rest either did not know (38; 61.3%) or chose the wrong answer (22; 35.5%). To determine the association between the course of study and knowledge of bioequivalence limit, the “I do not know” option was considered as an incorrect answer. The Kruskal–Wallis test showed no statistically significant difference (*p* = 0.545) among the three groups of final year undergraduate students with regard to their knowledge of the bioequivalence limit (Figure 1).

### 3.3. Knowledge and Perceptions about Generic Equivalent According to Course of Study

At least half of the final year students in all of the three faculties agreed that generic products of a particular medicine that are rated as generic equivalents are therapeutically equivalent to the innovator brand product. Additionally, almost all students in all three faculties agreed that they need more information on the conduct of bioequivalence tests. On the other hand, pharmacy (8/11, 72.8%) and nursing (9/9, 100%) students were more likely than their medical counterparts (20/42, 47.6%) to agree that generic products of a particular drug considered as generic equivalents are therapeutically equivalent to each other (*p* = 0.002). The details are highlighted in Table 2.

### 3.4. Knowledge and Perceptions of the Quality, Safety, and Efficacy of Generic Medicines versus Brand Name Drug According to Course of Study

With regard to quality, safety, and effectiveness of generic medicines compared to their branded equivalents, pharmacy (6/11, 54.5%) and nursing (6/9, 66.6%) students were likely to agree compared to their medical peers (13/42, 31.0%) that a generic medication is bioequivalent to its branded counterpart (*p* = 0.015). On the other hand, medical (29/42, 69.1%) and pharmacy (11/11, 100%) students were more likely to disagree than their nursing counterparts (5/9, 55.6%) that generic medicines are less effective than their originator brand product (*p* = 0.049). Additionally, medical (34/42, 81%) and pharmacy (9/11, 81.8%) students were more likely to disagree than nursing (2/9, 22.2%) students (*p* = 0.004) that generic medicines are of inferior quality to branded drugs. Surprisingly, less than half of medical (18/42, 42.9%), pharmacy (2/11, 18.2%), and nursing (3/9, 33.3%) students believed that generic medicines are less expensive than their branded counterparts. Although not statistically significant, pharmacy students (8/11, 72.7%) compared to their medical (23/42, 54.8%) and nursing (4/9, 44.4%) peers were against the notion that a generic medicine has more side effects than their corresponding innovator product. Additionally, (21/42, 50%), (5/11, 45.5%), and (6/9, 66.6%) of medical, pharmacy, and nursing students, respectively, agreed to a large extent that innovator brand drugs are required to meet higher safety standards than generic medicines (Table 3).

### 3.5. The Perceptions of Students about Generic Medicines According to Course of Study

Table 4 shows responses of all three cadres of healthcare students with regard to their perception of generic medicine. Based on their knowledge, medical and pharmacy students felt more comfortable to provide advice in the future with regard to generics medicines than their nursing counterparts (*p* < 0.001). Similarly, medical and pharmacy but nursing students were more likely to (*p* = 0.011) to agree that they found it easier to remember a medicine’s therapeutic class using generic names than its branded name. Additionally, medical and pharmacy students but nursing students believed that a pharmacist is the most appropriate person to advice on generic medicines. All of them agreed that they needed more information on issues about the safety and efficacy of generic medicines.

## 4. Discussion

The issue surrounding generic medicine use is of public health importance especially in developing countries where public access to essential medicines for common diseases is often hampered by the escalating cost of medication especially branded products [2]. The successful implementation of the generic medicine substitution policy in many countries around the world hinges on healthcare providers’ knowledge and perception of generic medicines [18]. Such an informed view will influence physician, pharmacist prescribing, and dispensing patterns, respectively. Our study results have identified a gap in knowledge and misconceptions about generics among these cohorts of future healthcare providers. It is hoped that our result will provide an opportunity for implementing educational interventions that address such knowledge deficit and misunderstanding about generic medications. Additionally, our findings will help inform the design of future generic medicine policy, regulations, and practice as well as direct the future research agenda with regard to generic medicine use in Sierra Leone.

The knowledge of the allowable regulatory bioequivalence limit among future healthcare professionals has been poor. The current research finding in this regard is not an exception as merely a couple of students chose the correct answer. This finding is in concordance with previously published studies in Australia, Nepal, Bangladesh, and Aruba [10,13,19,20]. This knowledge deficit exhibited by medical and nursing students was not surprising, as they are less likely to be exposed to the topics such as biopharmaceutics since their curricula are devoid of such a course [16]. However, it was surprising, even though it has been reported elsewhere [17], to learn that pharmacy students equally lack such knowledge given that they are expected to be educated on such a topic since it is part of their curriculum. A study in Yemen among pre-registration pharmacists indicated that they fully understood the concept of bioequivalence even with little exposure during their undergraduate training [21]. However, their overwhelming endorsement that they need more information on bioequivalence testing of generic medicines shows that the majority of medicine, pharmacy, and nursing students lack knowledge on bioequivalence of generic medicines and are unaware of the regulatory requirements for generic medicines approval in Sierra Leone. Such an observation may be explained in that, the concept of bioequivalence testing and the criteria for generic medicine approval in Sierra Leone are not part of the medicine and nursing curricula [16]. With regard to pharmacy students, it is possible that they might have been educated on the topic but with limited scope, or they might have failed to fully fathom the perceived difficult concepts of bioavailability and bioequivalence during their pharmacokinetics and pharmaceutics courses [22]. Either way, an introduction to such an important concept especially in medical and nursing curricula and proper teaching of such an important subject in the pharmacy program will help to address the observed knowledge gap. Improving these future healthcare providers’ knowledge on bioequivalence and bioequivalence testing of generic medicines will help boost their confidence in prescribing and dispensing generic medicines in the future. 

Students from all three groups agreed that all generic products of a particular drug referred to as generic equivalents are therapeutically equivalent to their innovator brand. A similar picture was reported by Hassali and colleagues [10]. On the other hand, our respondents agreed that generic equivalents of a particular innovator brand are therapeutically equivalent to each other. Our result does not correlate with what was reported in an Australian study where the majority of respondents disagreed that generic drugs considered as generic equivalents are therapeutically equivalents [10]. This difference is likely linked to the difference in regulatory policy regarding generic equivalents and its innovator brand. The drug registration guideline of the Pharmacy Board of Sierra Leone failed to state whether generics equivalents of a particular originator product should be therapeutically equal to each other. Instead, it states that data demonstrating that a generic medicine is bioequivalent to its innovator counterparts should be provided for market authorization of that a particular generic drug to be granted [23]. In Table 3, we observed that pharmacy students followed by nursing students were likely to believe, more than their medical counterparts, that a generic medicine and its corresponding innovator brand are bioequivalent to each other. This observed variation in perception among the three groups of students is likely linked to the difference in curricula. Subjects such as pharmaceutics that are extensively covered are not part of the current nursing and medical curricula at COMAHS-USL [16].

Regarding the safety quality and efficacy of generic medicines compared to their corresponding originator branded counterparts, pharmacy and medical students tend to have a better understanding compared to their nursing colleagues. For example, the majority of pharmacy and medical students compared to their nursing counterparts were against the notion that a generic drug is less effective and of low quality than its innovator product. On safety, a conflicting view was observed. On the one hand, most students in all three cadres (with nursing being the least) disagreed that generic medicines are less safe than their corresponding innovator brands. On the other hand, slightly more than half of the total respondents from all three groups (with nursing and pharmacy being the highest and least respectively) agreed that innovator brand name medicines require higher safety standards than their generic counterparts. Our result to a larger extent mirrors the result of a similar study conducted in Australia on medical and pharmacy students’ perception of efficacy quality and safety of generic medicines and their innovator brands [10]. However, it contrasts with studies done in Bangladesh [13] and Nigeria [11] in which pharmacy students considered generic medicines to be of low quality and less effective. This difference in views indicates that none of the group particularly medical and nursing cohorts had a clear understanding of the quality, efficacy, and safety requirements for generic medicines compared to their innovator brands in Sierra Leone [23]. It is also possible that variation in packaging and presentation may have influenced their varied perceptions. The negative perception of generic medicines especially among medicine and nursing students may lead to low faith in the use of generic medicines. For medical students, such a low confidence may negatively influence their future prescribing behavior of generic medicines if an educational intervention to change perception is not implemented. Thus, improving their knowledge through training will not only have a positive impact on the future prescribing and dispensing behavior but will also positively influence how these future healthcare providers educate their patients with regard to the use of generic medicines.

At most one-third (with pharmacy students being the least) agreed that generic medicines are less expensive than their branded name products. An appreciable number were not sure or chose to disagree. This is in sharp contrast to findings reported in Australia [10] and between pharmacy students in public and private universities in Pakistan in which the majority subscribed to the fact that generics are much cheaper than their branded counterparts [17]. The difference in view may be a function of their limited knowledge and misconception of issues surrounding generic medicines. This is primarily linked to little exposure to courses about generic medicines during their undergraduate training [16]. Additionally, their misconceptions that generic medicines are expensive compared to their innovator brands may be due to their inability to distinguish between certain generic medicines and their originator brands. The prices of certain generic medicines especially in the private sector are not regulated. Private drug companies solely set prices, and this may fluctuate based on the demand and supply of these products. The current national medicine policy is devoid of a specific policy statement on generic medicines including their pricing. It only encourages the use of generics in all public health facilities [4]. The overwhelming agreement by all final year undergraduate students in the three faculties that they needed more information on the quality and safety of generic medicines and that their healthcare training should include courses on national drug policy, essential medicine lists, and rational medicine use is a confirmation of their deficiency in knowledge and misconceptions about generic medicines. The use of an audio–visual educational package advocated in a Malaysian study [24] is also recommended to be adopted in the healthcare training institutions of Sierra Leone, as it is expected to critically appraise students about generics and their promotion in professional practice. Other educational interventions could include an interactive lecture that specifically covers topics such as bioequivalence, regulatory requirements for market authorization of generic medicine in Sierra Leone, generic medicine facts, and the promotion of rational prescribing and dispensing using generic medicines [25]. The distribution of reference materials to students such as a booklet that contains basic facts about generic medicines as well as a list of the generic names of commonly prescribed and dispensed medicines is also an educational intervention that helps improve knowledge among students [25]. 

A possible limitation of this study is the tendency for social desirability bias since questionnaires were self-administered. We also cannot exclude the possibility that participants might have discussed responses among themselves even though they were asked to answer the questions independently. Nevertheless, the major strength of this study is its high response rate, therefore, the conclusion derived from the study represents the views of final year undergraduate medicine, pharmacy, and nursing students in Sierra Leone. COMAHS-USL is the only tertiary healthcare training institution that offers undergraduate medicine, pharmacy, and nursing degree programs in Sierra Leone [26].

## 5. Conclusions

All three cadres of healthcare students, despite differences in responses, showed limited knowledge and misconceptions about generic medicines. In-depth training programs on issues surrounding generic drugs need to be designed and implemented by educators at COMAHS-USL.

## Figures and Tables

**Table 1:** Respondent demographics (*n* = 62).

Variables		Frequency	Percentages
Age Group	20–29 years	46	74.2
	30–39 years	16	25.8
Sex	Male	39	62.9
	Female	23	37.1
Program of study	Medicine	42	67.7
	Pharmacy	11	17.7
	Nursing	9	14.5

**Table 2:** Knowledge and perceptions about generic equivalent according to course of study.

Statements	Program of Study	SA *n* (%)	A *n* (%)	*N n* (%)	D *n* (%)	SD *n* (%)	*p*-Value ^1^
All generic products of a particular medicine that are rated as generic equivalents are therapeutically equivalent to the innovator brand product	Medicine *n* = 42	2 (4.8)	21 (50.0)	10 (23.8)	8 (19.0)	1 (2.4)	0.056
	Pharmacy *n* = 11	2 (18.2)	6 (54.5)	2 (18.2)	1 (9. 1)	0 (0.0)	
	Nursing *n* = 9	3 (33.3)	4 (44.4)	2 (22.2)	0 (0.0)	0 (0.0)	
	Total *n* (%)	7 (11.3)	31 (50.0)	14 (22.6)	9 (14.5)	1 (1.6)	
All generic products of a particular medicine that are rated as generic equivalents are therapeutically equivalent to each other	Medicine *n* = 42	1 (2.4)	19 (45.2)	9 (21.4)	12 (28.6)	1 (2.4)	0.002
	Pharmacy *n* = 11	3 (27.3)	5 (45.5)	1 (9.1)	2 (18.2)	0 (0.0)	
	Nursing *n* = 9	3 (33.3)	6 (66.7)	0 (0.0)	0 (0.0)	0 (0.0)	
	Total *n* (%)	7 (11.3)	30 (48.4)	10 (16.1)	14 (22.6)	1 (1.6)	
I have not been introduced to the issues of bioequivalence for generic drugs during my undergraduate education	Medicine *n* = 42	5 (11.9)	16 (38.1)	3 (7.1)	15 (35.7)	3 (7.1)	0.692
	Pharmacy *n* = 11	0 (0.0)	4 (36.4)	2 (18.2)	4 (36.4)	1 (9.1)	
	Nursing *n* = 9	0 (0.0)	3 (33.3)	2 (22.2)	4 (44.4)	0 (0.0)	
	Total *n* (%)	5 (8.1)	23 (37.1)	7 (11.7)	23 (37.1)	4 (6.5)	
I need more information on how bioequivalence tests are conducted for generic medicines	Medicine *n* = 42	18 (42.9)	22 (52.4)	1 (2.4)	1 (2.4)	0 (0.0)	0.237
	Pharmacy *n* = 11	3 (27.3)	7 (63.6)	1 (9.1)	0 (0.0)	0 (0.0)	
	Nursing *n* = 9	2 (22.2)	5 (55.6)	2 (22.2)	0 (0.0)	0 (0.0)	
	Total *n* (%)	23 (37.1)	34 (54.8)	4 (6.5)	1 (1.6)	0 (0.0)	

SA = strongly agree; A= agree; *n* = neutral; D = disagree; SD = strongly disagree, ^1^ Kruskall–Wallis test.

**Table 3:** Knowledge and perceptions of the quality, safety, and efficacy of generic medicines versus brand name medicine according to course of study.

Statements	Program of Study	SA *n* (%)	A *n* (%)	*N n* (%)	D *n* (%)	SD *n* (%)	*p*-Value ^1^
A generic medicine is bioequivalent to a brand name medicine	Medicine *n* = 42	1 (2.4)	12 (28.6)	15 (35.7)	14 (33.3)	0 (0.0)	0.015
	Pharmacy *n* = 11	1 (9.1)	5 (45.5)	3 (27.3)	2 (18.2)	0 (0.0)	
	Nursing *n* = 9	3 (33.3)	3 (33.3)	3 (33.3)	0 (0.0)	0 (0.0)	
	Total n (%)	5 (8.1)	20 (32.3)	21 (33.9)	16 (25.8)	0 (0.0)	
A generic medicine must be in the same dosage form (e.g., tablet, capsule) as the brand name medicine	Medicine *n* = 42	4 (9.5)	20 (47.6)	5 (11.9)	13 (31.0)	0 (0.0)	0.211
	Pharmacy *n* = 11	2 (18.2)	3 (27.3)	2 (18.2)	3 (27.3)	1 (9.1)	
	Nursing *n* = 9	1 (11.1)	7 (77.8)	1 (11.1)	0 (0.0)	0 (0.0)	
	Total n (%)	7 (11.3)	30 (48.4)	8 (12.9)	16 (25.8)	1 (1.6)	
A generic medicine must contain the same dose as the brand name medicine	Medicine *n* = 42	7 (16.7)	21 (50.0)	5 (11.9)	9 (21.4)	0 (0.0)	0.188
	Pharmacy *n* = 11	3 (27.3)	6 (54.5)	1 (9.1)	1 (9.1)	0 (0.0)	
	Nursing *n* = 9	2 (22.2)	7 (77.8)	0	0 (0.0)	0 (0.0)	
	Total *n* (%)	12 (19.4)	34 (54.8)	6 (9.7)	10 (16.1)	0 (0.0)	
Generic medicines are of inferior quality to branded drugs	Medicine *n* = 42	0 (0.0)	3 (7.1)	5 (11.9)	28 (66.7)	6 (14.3)	0.004
	Pharmacy *n* = 11	0 (0.0)	0 (0.0)	2 (18.2)	6 (54.5)	3 (27.3)	
	Nursing *n* = 9	0 (0.0)	1 (11.1)	6 (66.7)	2 (22.2)	0 (0.0)	
	Total *n* (%)	0 (0.0)	4 (6.5)	13 (21.0)	36 (58.1)	9 (14.5)	
Generic medicines are less effective than brand name medicines	Medicine *n* = 42	2 (4.8)	7 (16.7)	4 (9..5)	23 (54.8)	6 (14.3)	0.049
	Pharmacy *n* = 11	0 (0.0)	0 (0.0)	0 (0.0)	8 (72.7)	3 (27.3)	
	Nursing *n* = 9	0 (0.0)	1 (11.1)	3 (33.3)	5 (55.6)	0 (0.0)	
	Total *n* (%)	2 (3.2)	8 (12.9)	7 (11.3)	36 (58.1)	9 (14.5)	
Generic medicines are less safe than brand name medicines	Medicine *n* = 42	2 (4.8)	6 (14.3)	11 (26.2)	17 (40.5)	6 (14.3)	0.562
	Pharmacy *n* = 11	0 (0.0)	0 (0.0)	3 (27.3)	8 (72.7)	0 (0.0)	
	Nursing *n* = 9	0 (0.0)	1 (11.2)	4 (44.4)	4 (44.4)	0 (0.0)	
	Total *n* (%)	2 (3.2)	7 (11.3)	18 (29.0)	29 (46.8)	6 (9.7)	
Generic medicines are less expensive than brand name medicines	Medicine *n* = 42	7 (16.7)	11 (26.2)	15 (35.7)	6 (14.3)	3 (7.1)	0.330
	Pharmacy *n* = 11	2 (18.2)	0 (0.0)	5 (45.5)	3 (27.2)	1 (9.1)	
	Nursing *n* = 9	0 (0.0)	3 (33.3)	2 (22.2)	4 (44.4)	0 (0.0)	
	Total *n* (%)	9 (14.5)	14 (22.6)	22 (35.5)	13 (21.0)	4 (6.5)	
Brand name medicines are required to meet higher safety standards than generic medicines	Medicine *n* = 42	6 (14.3)	15 (35.7)	13 (31.0)	7 (16.7)	1 (2.4)	0.193
	Pharmacy *n* = 11	0	5 (45.5)	3 (27.2)	3 (27.2)	0 (0.0)	
	Nursing *n* = 9	3 (33.3)	3 (33.3)	3 (33.3)	0 (0.0)	0 (0.0)	
	Total *n* (%)	9 (14.5)	23 (37.1)	19 (30.6)	10 (16.1)	1 (1.6)	

SA = strongly agree; A= agree; N = neutral; D = disagree; SD = strongly disagree, ^1^ Kruskall–Wallis test.

**Table 4:** The perceptions of students about generic medicines according to course of study.

Statements	Program of Study	SA *n* (%)	A *n* (%)	*N n* (%)	D *n* (%)	SD *n* (%)	*p*-Value ^1^
From the knowledge I have, I’m confident in giving advice in the future by generic drug name rather than brand name	Medicine *n* = 42	9 (21.4)	27 (64.3)	4 (9.5)	2 (4.8)	0 (0.0)	<0.001
	Pharmacy *n* = 11	0 (0.0)	6 (54.5)	3 (27.3)	2 (18.2)	0 (0.0)	
	Nursing *n* = 9	0 (0.0)	0 (0.0)	6 (66.7)	3 (33.3)	0 (0.0)	
	Total *n* (%)	9 (14.5)	33 (53.2)	13 (21.0)	7 (11.3)	0 (0.0)	
I find it easier to recall a medicine’s therapeutic class using generic names rather than brand names	Medicine *n* = 42	20 (47.6)	17 (40.5)	2 (4.8)	3 (7.1)	0 (0.0)	0.011
	Pharmacy *n* = 11	3 (27.3)	5 (45.5)	2 (18.2)	1 (9.1)	0 (0.0)	
	Nursing *n* = 9	0 (0.0)	5 (55.6)	3 (33.3)	1 (11.1)	0 (0.0)	
	Total *n* (%)	23 (37.1)	27 (43.5)	7 (11.3)	5 (8.1)	0 (0.0)	
I believe that pharmacists are one of the most important health care professionals to give advice on generic medicines	Medicine *n* = 42	25 (59.5)	16 (38.1)	0 (0.0)	1 (2.4)	0 (0.0)	<0.001
	Pharmacy *n* = 11	7 (63.6)	4 (36.4)	0 (0.0)	0 (0.0)	0 (0.0)	
	Nursing *n* = 9	1 (11.1)	2 (22.2)	0 (0.0)	4 (44.4)	2 (22.2)	
	Total *n* (%)	33 (53.2)	22 (35.5)	0 (0.0)	5 (8.1)	2 (3.2)	
I believe that multinational products are of good quality than local company products	Medicine *n* = 42	8 (19.0)	18 (42.9)	12 (28.6)	2 (4.8)	2 (4.8)	0.282
	Pharmacy *n* = 11	2 (18.2)	4 (36.4)	4 (36.4)	1 (9.1)	0 (0.0)	
	Nursing *n* = 9	0 (0.0)	3 (33.3)	5 (55.6)	1 (11.1)	0 (0.0)	
	Total *n* (%)	10 (16.1)	25 (40.3)	21 (33.9)	4 (6.5)	2 (3.2)	
I need more information on the issues pertaining to the safety and efficacy of generic medicines	Medicine *n* = 42	17 (40.5)	22 (52.4)	1 (2.4)	1 (2.4)	1 (2.4)	0.764
	Pharmacy *n* = 11	3 (27.3)	8 (72.7)	0 (0.0)	0 (0.0)	0 (0.0)	
	Nursing *n* = 9	2 (22.2)	7 (77.8)	0 (0.0)	0 (0.0)	0 (0.0)	
	Total *n* (%)	22 (35.5)	37 (59.7)	1 (1.6)	1 (1.6)	1 (1.6)	
I believe my health training should include course on rational medicine use	Medicine *n* = 42	23 (54.7)	17 (40.5)	1 (2.4)	0 (0.0)	1 (2.4)	0.110
	Pharmacy *n* = 11	6 (54,5)	5 (45.5)	0 (0.0)	0 (0.0)	0 (0.0)	
	Nursing *n* = 9	2 (22.2)	5 (55.6)	2 (22.2)	0 (0.0)	0 (0.0)	
	Total *n* (%)	31 (50.0)	27 (43.5)	3 (4.8)	0 (0.0)	1 (1.6)	
I believe my health training should include course on national drug policy and essential drug list	Medicine *n* = 42	28 (66.7)	13 (30.9)	0 (0.0)	0 (0.0)	1 (2.4)	0.208
	Pharmacy *n* = 11	6 (54,5)	5 (45.5)	0 (0.0)	0 (0.0)	0 (0.0)	
	Nursing *n* = 9	3 (33.3)	6 (66.7)	0 (0.0)	0 (0.0)	0 (0.0)	
	Total *n* (%)	37 (59.7)	24 (38.7)	0 (0.0)	0 (0.0)	1 (1.6)	

SA = strongly agree; A= agree; N = neutral; D = disagree; SD = strongly disagree, ^1^ Kruskall–Wallis test.

## Abbreviations

PBSL	Pharmacy Board of Sierra Leone
COMAHS-USL	College of Medicine and Allied Health Sciences, University of Sierra Leone
